# Dynamic coupling between the central and autonomic cardiac nervous systems in patients with refractory epilepsy: A pilot study

**DOI:** 10.3389/fneur.2022.904052

**Published:** 2022-08-10

**Authors:** Eline Melo, José Fiel, Rodrigo Milhomens, Thaynara Ribeiro, Raphael Navegantes, Francinaldo Gomes, Bruno Duarte Gomes, Antonio Pereira

**Affiliations:** ^1^Graduate Program in Neuroscience and Cell Biology, Federal University of Pará, Belém, Brazil; ^2^Graduate Program in Electrical Engineering, Federal University of Pará, Belém, Brazil; ^3^Department of Electrical and Biomedical Engineering, Institute of Technology, Belém, Brazil; ^4^Hospital Ophir Loyola, Belém, Brazil; ^5^Department of Biotechnology, Institute of Biological Sciences, Federal University of Pará, Belém, Brazil

**Keywords:** refractory epilepsy, heart rate variability (HRV), EEG, heartbeat evoked potential, autonomic nervous system (ANS)

## Abstract

The heart and brain are reciprocally interconnected and engage in two-way communication for homeostatic regulation. Epilepsy is considered a network disease that also affects the autonomic nervous system (ANS). The neurovisceral integration model (NVM) proposes that cardiac vagal tone, indexed by heart rate variability (HRV), can indicate the functional integrity of cognitive neural networks. ANS activity and the pattern of oscillatory EEG activity covary during the transition of arousal states and associations between cortical and autonomic activity are reflected by HRV. Cognitive dysfunction is one of the common comorbidities that occur in epilepsy, including memory, attention, and processing difficulties. Recent studies have shown evidence for the active involvement of alpha activity in cognitive processes through its active role in the control of neural excitability in the cortex through top-down modulation of cortical networks. In the present pilot study, we evaluated the association between resting EEG oscillatory behavior and ANS function in patients with refractory epilepsy. Our results show: (1) In patients with refractory epilepsy, there is a strong positive correlation between HRV and the power of cortical oscillatory cortical activity in all studied EEG bands (delta, theta, alpha, and beta) in all regions of interest in both hemispheres, the opposite pattern found in controls which had low or negative correlation between these variables; (2) higher heartbeat evoked potential amplitudes in patients with refractory epilepsy than in controls. Taken together, these results point to a significant alteration in heart-brain interaction in patients with refractory epilepsy.

## Introduction

Epilepsy affects more than 70 million people worldwide (1) and about 30–40% of these patients have drug-resistant or refractory epilepsy (RE) (2). The International League Against Epilepsy (ILAE) defines drug-resistant epilepsy as “failure of adequate trials of two tolerated, appropriately chosen and used antiepileptic drug schedules (whether as monotherapies or in combination) to achieve sustained seizure freedom” (3). The *Neural Network Hypothesis* proposes that seizure-induced degeneration and remodeling of the neural network suppresses the brain's seizure control system and restricts antiseizure drugs from accessing neuronal targets (4). However, it seems these changes are not confined to cortical and subcortical structures, they also compromise the functioning of the Autonomic Nervous System (ANS) (5), which regulate the function of various organs and systems, such as the cardiovascular system (6).

The brain and heart are reciprocally interconnected and engage in two-way communication for homeostatic regulation (7, 8). This relationship is also important for the control of cognitive and emotional processes, as proposed by the neurovisceral integration model (NVM) (9), which states that capacity of adaptation to unexpected changes in the environment is directly related to biological flexibility within the neural network regulating autonomic responses. A key parameter indexing neurocardiac function is heart rate variability (HRV) (7, 10).

Heart rate variability (HRV) is the fluctuation in the time intervals between adjacent heartbeats and is supposed to reflect vagally-mediated brain influence (11–14). Lower HRV has been linked to reduced selfregulatory capacity and negative effects on cognitive functions that involve the executive centers of the prefrontal cortex (12). HRV can be evaluated with both time- and spectral-domain metrics [for review, see (13)]. The following four metrics have been extensively used for assessment of HRV in the time-domain: SDNN (Standard deviation of NN intervals), SDANN (Standard deviation of the average NN intervals for each 5 min segment of a 24 h HRV recording), and RMSSD (Root mean square of successive RR interval differences) (13). In the frequency domain, the three metrics of choice are low frequency (LF) power (0.04–0.15 Hz), high frequency (HF) power (0.15–0.40 Hz), and the LF/HF ratio (15).

In the present work, we compare the function of the central autonomous network of patients with refractory epilepsy to a group of age-related control subjects. We verify the association between HRV measures and frequency-domain parameters of resting-state EEG recordings and also evaluate how the heartbeat evoked potential, a measure of cortical responsivity to heartbeats (16), of people with refractory epilepsy might be different from neurotypicals. Our hypothesis is that central autonomous networks may be altered in people with epilepsy, and this could contribute to strength the view that epilepsy results in the alteration of widespread neural networks including the central nervous system.

## Materials and methods

The participants of the present study were divided into two groups, the control group, comprised of 7 subjects (34.4 ± 9.9 years old; 4 males and 3 females), and the refractory epilepsy group (RE), comprised of 11 patients (32.3 ± 10.2 years old; 6 males and 5 females). Inclusion criteria were: Diagnosed refractory epilepsy. Subjects in the control group must not have presented a history of diagnosed neurological disease, as well as not being taken medicines for neurological diseases, or that might cause cognitive impairment while the research was conducted. The exclusion criteria were: Regular use of alcohol, cigarettes, or other addictive substances; Presence of Sleep-related breathing disorders; Presence of significant progressive disorders or unstable medical conditions requiring acute intervention; Change in AED regimen in the last 28 days; Subject is currently taking >3 concomitant AEDs; Subject has had status epilepticus within the past 2 years; A psychiatric disorder where changes in pharmacotherapy are needed or anticipated during the study; Any condition that may impact a subject's ability to follow study procedures or subject's safety, based on what is known about the pharmacology/toxicology profile of the trial agent(s); Time of onset of epilepsy treatment <2 years prior to enrollment.

All subjects underwent simultaneous EEG and ECG recordings while they were at rest. The procedure was task free. The subjects were invited to sit down in a comfortable chair located in a silent room with well-controlled illumination and temperature. The total duration of each recording session was 10 min, the first 9 min were with eyes opened and the last minute with eyes closed. During the session, the subjects stared at a blank wall located 1.4 m ahead.

EEG signals were collected using a 22 channel Neuromap 40i system (Neurotec, Brazil) with Ag/AgCl electrodes placed according to the 10/20 electrode placement standard. EEG data was sampled at 256 Hz and the electrode impedances were kept under 10 kΩ throughout the experiment. The Fpz and auricular electrodes were used as ground and reference, respectively. The ECG signals were collected with the same device used for the EEG recordings (Neuromap 40i) *via* a bipolar configuration using the patients' upper limbs (peripheral derivation). The sampling rate was 256 Hz and the ground electrode was located at Fpz.

### Preprocessing and spectral analysis of EEG data

During EEG recordings, the signal was band-pass filtered between 0.5 and 100 Hz with a Butterworth filter. A notch filter centered at 50 Hz was used for noise suppression. The preprocessing routine was performed using Matlab (Mathworks, Inc) and the free toolbox EEGLAB (17). EEG channels were re-referenced to the linked mastoids. Ocular and muscle artifacts were removed using visual inspection and through independent component analysis (ICA) (18).

EEG signals were decomposed in their constituent frequency bands using the fast Fourier transform (FFT) with a Hamming window over the recording period. The frequency ranges selected were delta (d: 1–4 Hz), theta (q: 4–8 Hz), alpha 1 (a1: 8–10 Hz), alpha 2 (a2: 10–13 Hz), and beta (b: 13–31 Hz). For each frequency range the average power was calculated for five regions of interest (ROI) corresponding to electrode channels in the frontal, central, temporal, parietal, and occipital lobes, respectively. Average power X_m_ of EEG sensors were calculated with the Welch's method (averaged periodogram) with 0% overlap and expressed in decibels according to the relation X_m_ = 10log10 (mV^2^/Hz).

### Heart rate variability analysis

The heart rate variability (HRV) was analyzed in the time and frequency domains according to the recommended standards for HRV measurement (15). The HRV analysis was performed using the software Kubios (19). Time-domain analysis provided the following variables: SDNN (standard deviation of the beat-to-beat or NN intervals), RMSSD (root mean square of successive differences between normal heartbeats), and SD1, which corresponds to a dynamical non-linear analysis reflecting the variability of beat-to-beat. RMSSD and SD1 are identical metrics of HRV (20) and reflect parasympathetic vagal tone (21). FFT was used to extract the power of NN intervals corresponding to 0.15–0.4 Hz and obtain the Hear rate frequency (HF).

### Association between brain and cardiac activity

For an intragroup analysis of the association between brain and heart activity, we used a Spearman correlation to compare HRV parameters (SDNN, RMSSD, SD1) with the mean power of each frequency range from the EEG. The *p*-value was used as a measure of the probability that any observed correlation was due to chance. We considered a significance level of 0.05 for this aim, cortical regions of interest (ROI) were set as follows: Frontal left hemisphere (Fp1, F3, F7), frontal right hemisphere (Fp2, F4, F8); Central (C3, C4); Temporal left hemisphere (T3, T5), temporal right hemisphere (T4, T6); Parietal left and right hemisphere, P3 and P4, respectively; and occipital left and right hemispheres, O1 and O2, respectively. Furthermore, a comparison between EEG power and HRV parameters between groups was performed using one-way ANOVA (a = 0.05).

For each group, multiple linear regression models were calculated with EEG channel's power as the independent variables and the HRV parameters as the dependent variables. That is, for each frequency band, a given HRV parameter was set as a dependent variable, and the power in each of the five ROI's (F, C, P, T, O) was set as the independent variable such that the probabilistic functional below could be evaluated:


$$\begin{array}{l} \text{HRV }\approx \;\alpha \text{F}+\beta \text{C}+\delta \text{P}+\varepsilon \text{T}+\phi \text{O} \end{array}$$

The coefficients (a, b, e, f) were estimated using least squares solved by QR decomposition using Matlab (*Mathworks*). For each linear model, multiple regressions were performed after removing predictors to check for those that better explained the variability of the HRV parameter. The adjusted *R*^2^ was used to indicate which of the five predictors (power at F, C, P, T, and O) better contributed to explaining the variation of each of the HRV parameters (HF, RMSSD, SDNN). The distance between observed and expected values of the regression was evaluated using Pearson residuals. The Shapiro-Wilk test was used to check if the residuals follow a normal distribution at a 5% significance level.

### Heartbeat-evoked potentials

ECG data was epoched around each identified R peak (200 ms before the R-peak to 600 ms following it) and was low-passed filtered at 15 Hz. Since the cardiac-field artifacts often interfere with HEP analysis, we chose to analyze event-related potentials occurring within a time window of interest (TOI) of 350–600 ms across EEG electrodes to ensure that all evoked components reported in the literature were present (22). The HEP for both groups were averaged from 179 trials. All the analysis was performed using EEGLAB and Matlab (Mathworks, Inc.).

## Results

**Figure 1:**
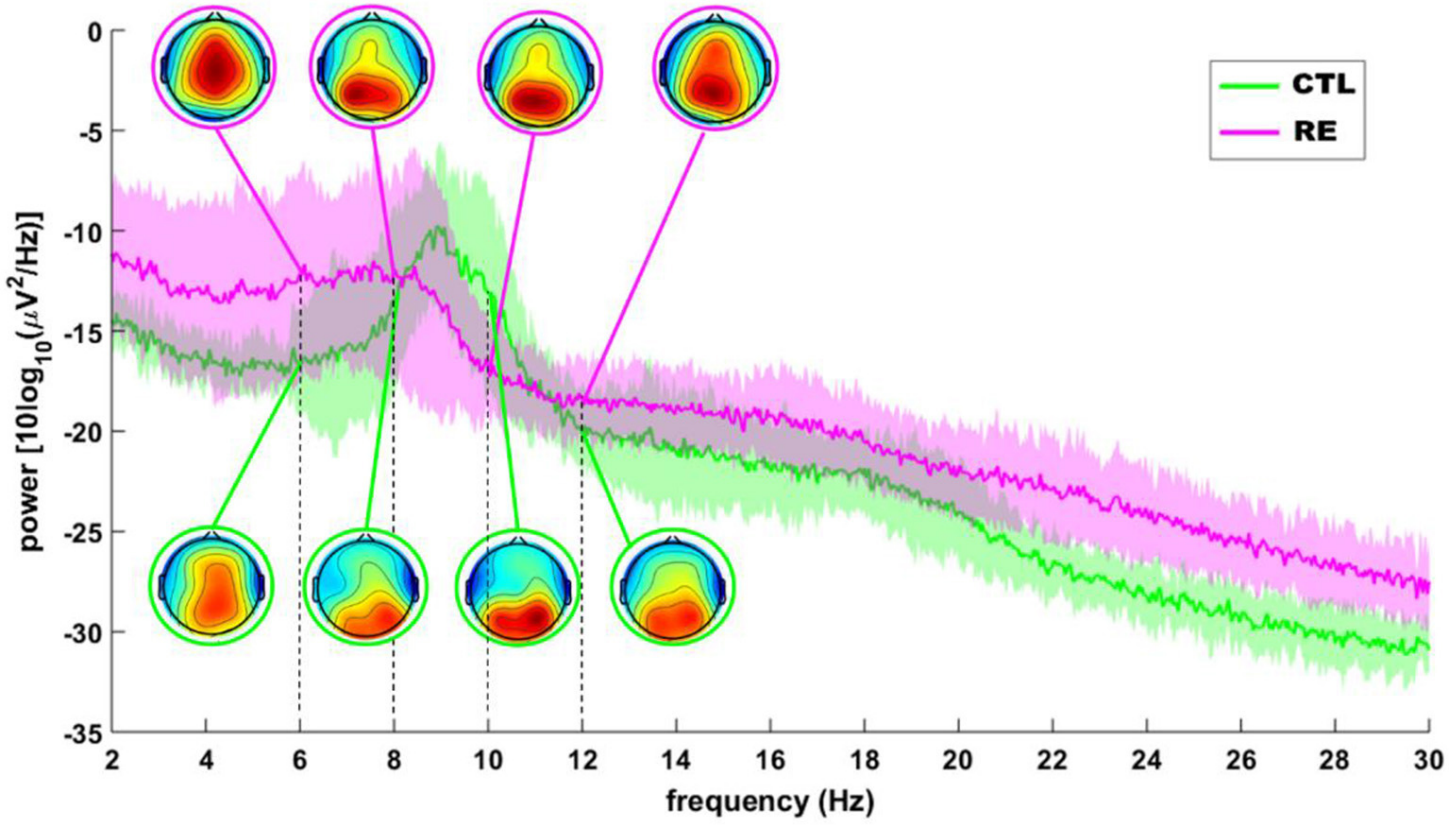
Grand average of the EEG power spectrum of RE (red) and control (green) subjects. Insets depict, topographical distribution of mean alpha power. The shaded area around each line is the SD.

Table 1 shows the clinical data for each subject of the RE group. There was no difference between the two experimental groups regarding the following HRV parameters after Bonferroni correction for multiple comparison: SDNN (*p* = 0.97); RMSSD (*p* = 1.00); SD1 (*p* = 1.00) and HF (*p* = 1.00). Also, there was no difference between the experimental groups regarding any frequency band in any ROI after Bonferroni correction for multiple comparison: Delta (Frontal, *p* = 1.00; Central, *p* = 0.35; Temporal, *p* = 0.80; Parietal, *p* = 1.00; Occipital, *p* = 1.00), Theta (Frontal, *p* = 1.00; Central, *p* = 1.00; Temporal, *p* = 1.00; Parietal, *p* = 1.00; Occipital, *p* = 1.00), Alpha-1 (Frontal, *p* = 0.35; Central, *p* = 0.40; Parietal, *p* = 0.70; Temporal, *p* = 1.00; Occipital, *p* = 1.00), Alpha-2 (Frontal, *p* = 0.30; Central, *p* = 1.00; Temporal, *p* = 1.00; Parietal, *p* = 1.00; Occipital, *p* = 1.00), Beta (Frontal, *p* = 0.30; Central, *p* = 0.70; Temporal, *p* = 0.85; Occipital, *p* = 1.00). However, while the average power spectrum of the control group had an alpha peak around 9 Hz, that of the RE group was not as clear and seemed to shift to the left (Figure 1).

**Table 1:** Clinical data for the evaluated patients.

**Patient**	**Age**	**Gender**	**Seizures onset (Age)**	**Epilepsy type/Cause**	**AEDs (mg/day)**	**Seizures frequency (month)**	**Seizure type**
1	33	M	7	TLE L	CBZ/1,200; PHB/120	12	Focal
2	45	M	22	TLE L/Cavernoma	PHB/100; PTH/300	4	Focal
3	17	F	2	TLE B/FCD	CBZ/1,200, CLM/200, PHB/200	+100	Generalized
4	24	F	12	TLE L/PNET	PHB/200, PTH/ 100	5	Focal
5	45	M	24	TLE R/Gliosis	CBZ/1,200, SVP/1,500, TPM/200	8	Focal
6	31	F	14	FLE B	CLM/20. PTH/300, SVP/1,500	12	Generalized
7	35	M	6	TLE L	CBZ/1,000, PHB/100	12	Focal
8	49	M	45	Unknown	PTH/100	4	Generalized
9	26	M	0	Schizencephaly R	PTH/300, SVP/1,500, CLM/20	+100	Generalized
10	24	F	2	TLE B	CBZ/900, PHB/100	12	Focal
11	27	F	4	TLE R	SVP/1,500, CBZ/20	12	Focal

Gender: M, Male; F, Female; Epilepsy type/cause: TLE, Temporal lobe epilepsy; FCD, Focal cortical dysplasia; PNET, Primitive neuroectodermal tumor; FLE, Frontal lobe epilepsy; R, Right; L, Left; B, Bilateral; AEDs, Antiepileptic drugs; SVP, Sodium valproate; CBZ, Carbamazepine; PTH, Phenytoin; PHB, Phenobarbital; TMP, Topiramate; CLM, Clobazam.

**Figure 2:**
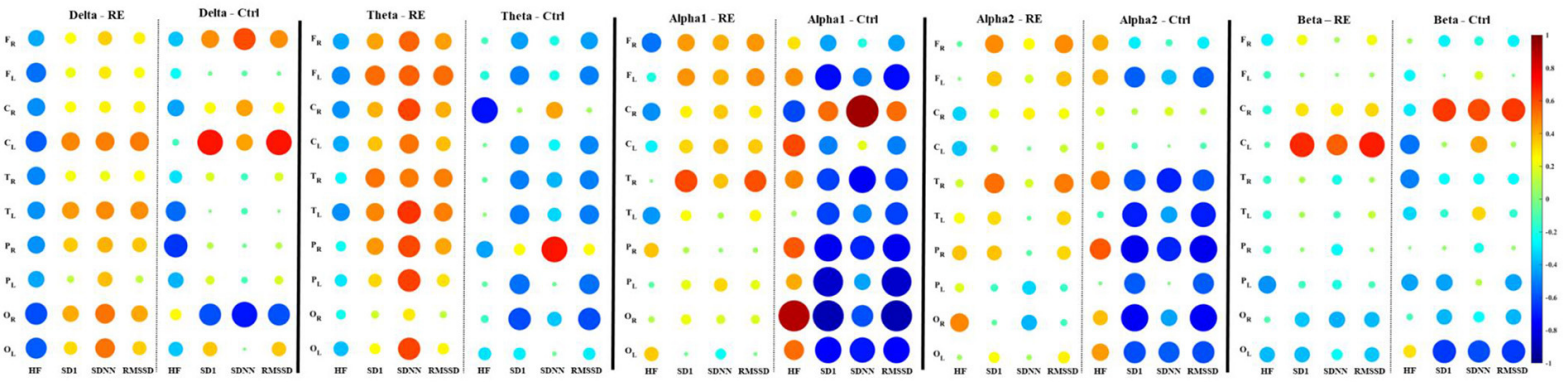
Spearman correlation correlograms between EEG average power of frequency bands (Delta, theta, apha1, apha2, and beta) and HRV parameters for the refractory epilepsy (RE) and control (Ctrl) groups. In each correlogram, the HRV parameters correspond to columns and the rows correspond to the ROI from which the mean power was calculated (F, frontal; C, central; T, temporal; P, parietal; O, occipital). The subscripts R or L indicate the right and left cerebral hemispheres, respectively. Correlation values are indicated by color according to the scale bar on the right. The correlation values correspond to the radius of the discs.

The intragroup correlations between EEG power and HRV parameters for the RE and control groups are shown in Tables 2, 3, respectively. We chose to show just the correlations that were equal to or above 0.5 with a significant *p*-value (*p* < 0.05). While typically positive correlations were found for the RE group (Table 2), the control group had most negative correlations (Table 3). Specifically, in the RE group, activity in the theta frequency range correlated positively with SD1/RMSSD, HF, and SDNN parameters in every ROI in both hemispheres. Alpha was correlated positively with SD1/RMSSD mostly in the temporal lobe in the left hemisphere. Beta frequency was positively correlated with SD1/RMSSD and HF in the frontal lobe of the right and left hemisphere, respectively. The negative correlations found for the control group were mostly restricted to activity in the alpha frequency band and only for SD1/RMSSD in every lobe in both hemispheres (Table 3). The correlograms comprising all the estimated correlations can be seen in Figure 2.

**Table 2:** Spearman correlations calculated between EEG power and HRV parameter for the refractory epilepsy group.

**Correlations**	**p**	**Group**	**Hemisphere**	**C.L**	**HRV-Par**	**EEG-F**
0.62	0.048	RE	L	C	SDNN	Theta
0.63	0.044	RE	R	T	SDNN	Theta
0.64	0.037	RE	R	F	SDNN	Theta
−0.65	0.030	RE	L	F	HF	Theta
−0.66	0.027	RE	R	F	HF	Theta
0.66	0.031	RE	L	T	RMSSD	Theta
0.68	0.025	RE	L	T	SDNN	Theta
0.68	0.020	RE	L	T	SD1	Theta
0.69	0.019	RE	R	O	SD1	Theta
0.70	0.021	RE	R	O	RMSSD	Theta
0.73	0.021	RE	R	P	SDNN	Theta
0.83	0.003	RE	R	O	SDNN	Theta
−0.65	0.046	RE	L	F	HF	Alpha 1
0.67	0.039	RE	R	C	RMSSD	Alpha 1
0.74	0.012	RE	L	T	RMSSD	Alpha 1
0.76	0.006	RE	L	T	SD1	Alpha 1
0.73	0.015	RE	L	T	RMSSD	Alpha 2
0.75	0.007	RE	L	T	SD1	Alpha 2
0.67	0.023	RE	R	F	SD1	Beta
0.70	0.021	RE	R	F	RMSSD	Beta
−0.77	0.010	RE	L	F	HF	Beta

R, right; L, left; C.L, Cortical lobe; HRV-Par, Heart rate variability parameter; EEG-F, EEG frequency range.

**Table 3:** Spearman correlations calculated between EEG power and HRV parameter for the control epilepsy group.

**Correlations**	**p**	**Group**	**Hemisphere**	**C.L**	**HRV-Par**	**EEG-F**
−0.82	0.034	CTL	R	F	RMSSD	Theta
−0.82	0.034	CTL	R	F	SD1	Theta
−0.82	0.034	CTL	R	C	RMSSD	Theta
−0.82	0.034	CTL	R	C	SD1	Theta
−0.86	0.024	CTL	L	T	SDNN	Theta
−1.00	0.000	CTL	R	F	RMSSD	Alpha 1
−1.00	0.000	CTL	R	F	SD1	Alpha 1
−0.78	0.048	CTL	L	P	RMSSD	Alpha 1
−0.78	0.048	CTL	L	P	SD1	Alpha 1
−0.82	0.034	CTL	L	T	SDNN	Alpha 1
−0.86	0.024	CTL	R	P	RMSSD	Alpha 1
−0.86	0.024	CTL	R	P	SD1	Alpha 1
−0.86	0.024	CTL	R	T	RMSSD	Alpha 1
−0.86	0.024	CTL	R	T	SD1	Alpha 1
0.94	0.017	CTL	L	C	SDNN	Alpha 1
−1.00	0.000	CTL	R	F	RMSSD	Alpha 2
−1.00	0.000	CTL	R	F	SD1	Alpha 2
−0.78	0.048	CTL	L	P	RMSSD	Alpha 2
−0.78	0.048	CTL	L	P	SD1	Alpha 2
−0.89	0.033	CTL	L	T	RMSSD	Alpha 2
−0.89	0.033	CTL	L	T	SD1	Alpha 2

R, right; L, left; C.L, Cortical lobe; HRV-Par, Heart rate variability parameter; EEG-F, EEG frequency range.

Tables 4, 5 show the main parameters of the multiple linear regressions for each EEG frequency range and the corresponding HRV variable and cortical ROI. The maximum value of adjusted *R*^2^ was obtained with the frontal and central cortical areas as predictors of HRV variables. For the RE group, and in contrast to the results of the Spearman correlation, only theta power showed to be a good predictor for HRVs parameters. That was the case for RMSSD regressed with the power of all EEG channels of the right hemisphere, and for SDNN for both the left and right hemispheres, the latter having all EEG channels as predictors except for the occipital region (Table 4). On the other hand, the same analysis for the control group showed that the power of all frequency ranges were good predictors of HRV variables with high values of adjusted *R*^2^, markedly the theta and alpha 1 and 2 ranges (Tables 4, 5).

**Table 4:** Multiple regression parameters for the HRV variables as a function of EEG power (delta, theta, and alpha 1).

**Group**	**Hemisphere**		**Delta**			**Theta**			**Alpha 1**	
			***R* ^2^**			***R* ^2^**			***R* ^2^**	
		**HF**	**RMSSD**	**SDNN**	**HF**	**RMSSD**	**SDNN**	**HF**	**RMSSD**	**SDNN**
Control	L	0.880	0.372	0.182	0.932	0.954	0.990	0.606	0.997	0.990
	R	0.974	0.934	0.992	0.318	0.534	0.485	0.419	0.979	0.853
RE	L	0.219	0.055	0.549	0.308	0.748	0.818	0.029	0.497	0.368
	R	0.755	0.635	0.244	0.230	0.890	0.735	0.093	0.310	0.376
			***R*^2^ (adj)**			***R*^2^ (adj)**			***R*^2^ (adj)**	
Control	L	0.279	0.058	−0.226	**0.797**	**0.909**	**0.998**	0.409	**0.981**	**0.943**
	R	**0.922**	**0.604**	0.950	−0.023	0.300	0.228	0.129	**0.877**	**0.559**
RE	L	0.024	−0.181	0.248	0.011	0.497	**0.635**	−0.213	0.162	0.097
	R	0.510	0.269	0.055	0.037	**0.780**	**0.558**	−0.134	0.015	0.108
			**SE**			**SE**			**SE**	
Control	L	F (0.171)	F (5.183)	F (6.976)	**F (0.042)**	**F (5.100)**	**F (1.003)**	F (0.018)	**F (0.822)**	**F (1.674)**
		C (0.360)	C (8.179)	C (11.008)	**C (0.026)**	**C (3.151)**	**C (0.604)**	C (0.020)	**C (0.863)**	**C (1.757)**
		P (0.112)			**P (0.046)**	**P (5.498)**	**P (1.509)**		**P (0.693)**	**P (1.412)**
		T (0.171)			**T (0.011)**		**T (0.268)**		**T (0.405)**	**T (0.826)**
		O (0.105)					**O (0.321)**		**O (0.575)**	**O (1.171)**
	R	**F (0.012)**	**F (5.521)**	F (2.321)	F (0.034)	F (5.118)	F (6.343)	F (0.015)	**F (2.305)**	**F (5.140)**
		**C (0.013)**	**C (7.790)**	C (3.276)	C (0.020)	C (2.976)	C (3.688)	C (0.018)	**C (2.544)**	**C (5.658)**
		**P (0.018)**	**P (9.609)**	P (4.041)					**P (1.709)**	**P (3.161)**
		**T (0.009)**	**T (4.197)**	T (1.765)					**T (3.464)**	**T (6.499)**
			**O (3.083)**	O (1.297)					**O (1.517)**	
RE	L	F (0.011)	F (5.745)	F (3.920)	F (0.011)	F (5.684)	**F (4.040)**	F (0.007)	F (4.694)	F (2.472)
		C (0.008)	C (4.117)	C (3.349)	C (0.010)	C (4.216)	**C (2.997)**	C (0.005)	C (2.263)	C (1.846)
				P (2.593)	P (0.016)	P (5.913)	**P (4.203)**		P (4.289)	P (2.357)
				T (2.043)		T (3.575)	**T (2.541)**		T (3.580)	
						O (2.654)	**O (1.886)**			
	R	F (0.020)	F (11.314)	F (7.356)	F (0.011)	**F (3.102)**	**F (3.511)**	F (0.009)	F (4.334)	F (3.444)
		C (0.015)	C (8.579)	C (4.997)	C (0.013)	**C (3.461)**	**C (4.086)**	C (0.011)	C (5.149)	C (4.092)
		P (0.012)	P (6.606)			**P (1.967)**	**P (2.326)**		P (2.535)	P (2.015)
		T (0.009)	T (4.959)			**T (2.463)**	**T (2.469)**			
		O (0.011)	O (5.964)			**O (2.359)**				

RE, refractory epilepsy; L, left; R, right; SE, standard error; F, frontal; C, central; P, parietal; T, temporal; O, occipital. Bold values indicate adjusted R-squared higher than 0.5 and the corresponding standard error values for each regression predictor.

**Table 5:** Multiple regression parameters for HRV variables as a function of EEG power (alpha 2 and beta).

**Group**	**Hemisphere**		**Alpha 2**			**Beta**	
			***R* ^2^**			***R* ^2^**	
		**HF**	**RMSSD**	**SDNN**	**HF**	**RMSSD**	**SDNN**
Control	L	0.946	0.974	0.883	0.91	0.45	0.22
	R	0.306	0.963	0.926	0.2	0.96	0.97
RE	L	0.006	0.549	0.348	0.24	0.33	0.18
	R	0.067	0.625	0.565	0.3	0.16	0.17
			***R*^2^ (adj)**			***R*^2^ (adj)**	
Control	L	**0.839**	**0.844**	**0.824**	**0.82**	−0.11	−0.18
	R	−0.041	**0.775**	**0.554**	−0.2	**0.92**	**0.91**
RE	L	−0.242	0.248	−0.087	−0.09	0.16	−0.02
	R	−0.166	0.251	0.131	0	−0.06	−0.04
			**SE**			**SE**	
Control	L	**F (0.007)**	**F (1.730)**	**F (1.485)**	**F (0.006)**	F (2.781)	F (3.365)
		**C (0.007)**	**C (2.316)**	**C (1.510)**	**C (0.006)**	C (2.763)	C (3.037)
		**P (0.008)**	**P (2.068)**		**P (0.011)**	P (5.294)	
		**T (0.006)**	**T (1.090)**				
			**O (2.787)**				
	R	F (0.015)	**F (2.871)**	**F (4.775)**	F (0.017)	**F (0.835)**	**F (1.505)**
		C (0.019)	**C (3.567)**	**C (5.931)**	C (0.018)	**C (0.955)**	**C (1.297)**
			**P (2.588)**	**P (4.304)**		**P (1.901)**	**P (3.415)**
			**T (2.496)**	**T (4.151)**			**T (1.544)**
			**O (1.983)**	**O (3.298)**			
RE	L	F (0.006)	F (3.567)	F (3.582)	F (0.006)	F (2.396)	F (2.202)
		C (0.005)	C (1.915)	C (1.923)	C (0.004)	C (1.697)	C (1.560)
			P (4.687)	P (4.706)	P (0.007)		
			T (3.293)	T (3.307)			
							
	R	F (0.007)	F (4.153)	F (3.736)	F (0.011)	F (2.979)	F (2.465)
		C (0.009)	C (7.202)	C (6.479)	C (0.009)	C (3.126)	C (2.587)
			P (5.671)	P (5.101)	P (0.010)		
			T (5.576)	T (5.016)			
			O (8.045)	O (7.237)			

RE, refractory epilepsy; L, left; R, right; SE, standard error; F, frontal; C, central; P, parietal; T, temporal; O, occipital). Bold values indicate adjusted R-squared higher than 0.5 and the corresponding standard error values for each regression predictor.

**Figure 3:**
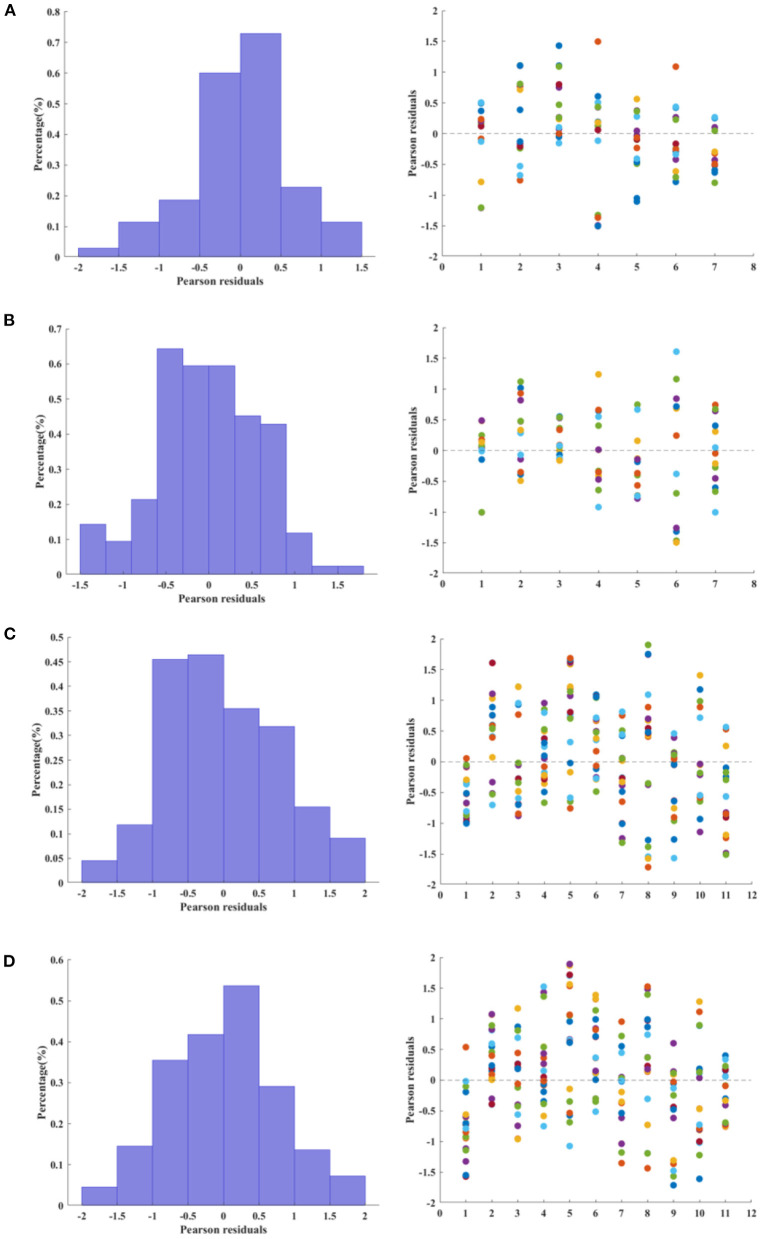
Relative frequency (left column) and scatter plot (right column) of Pearson residuals estimated from multiple linear regression. For the histograms, the relative frequency was normalized as a probability density function, i.e., the area of each bar is the relative number of residuals observed for a given interval and the sum of the bar areas is less than or equal to one. **(A,B)** Control group, left and right hemisphere, respectively. **(C,D)**. Refractory epilepsy group, left and right hemisphere, respectively.

The distribution and scatter plot of Pearson residuals are shown in Figure 3. Only for the RE group (right hemisphere) the null hypothesis that the residuals follow a normal distribution was rejected (Shapiro-Wilk test, *p* = 0.0246).

**Figure 4:**
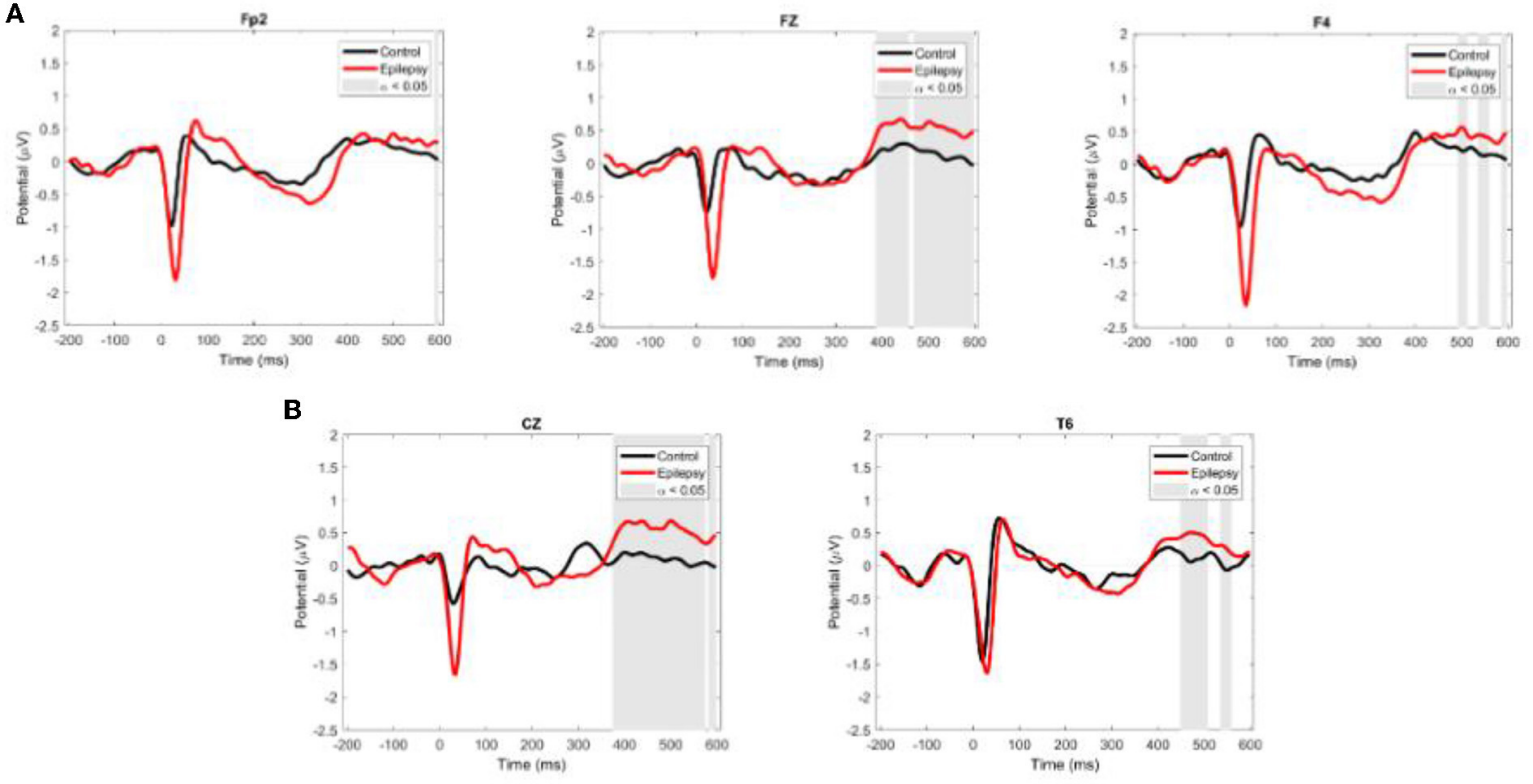
Grand average ERP waveforms for EEG channels with intergroup differences. **(A)** Channels where HEP amplitude is higher in the RE than in the control group. **(B)** Channels where HEP amplitude is higher in the control than in the RE group. Red and black lines are ER and control values, respectively. Shaded areas show the time-windows where there was a significant difference between groups (ANOVA, a = 0.05).

HEPs had a larger amplitude in the RE than in the control group in the following channels (with time window of significant activation after R peak and corrected for multiple comparisons with Bonferroni): Fp2 (597–656 ms, *p* = 0.04), Fz (597–656 ms, *p* = 0.04) and 597–656 ms (*p* = 0.04), F3 (484–554 ms, *p* = 0.01; 578–656 ms, *p* = 0. 01), F4 (468–492 ms, *p* = 0.02; 597–656 ms, *p* = 0.03), F8 (589–656 ms, *p* = 0.01), Cz (375–597 ms, *p* = 0.01), T6 (449–503 ms, *p* = 0.01; 535–554 ms, *p* = 0.02) (Figure 4).

## Discussion

Disorders of generalized arousal underlie many problems in motivated behavioral responses, cognitive functions, and emotional expression (23). One valuable tool for assessing arousal is the spectral analysis of the resting-state human electroencephalogram (EEG). Changes in the power of the EEG frequency bands during wakefulness are closely related to alterations in arousal. For instance, alterations of the level of vigilance are reflected, essentially, in topographical changes in the activity of slow waves, such as alpha (24).

Fluctuations in arousal also reflect the activity of the autonomous nervous system (ANS), which is controlled by the balanced activity of the sympathetic and parasympathetic systems. The neurovisceral integration model (9) proposes that cardiac vagal tone, indexed by heart rate variability (HRV), can indicate the functional integrity of cognitive neural networks (25). ANS activity and the pattern of oscillatory EEG activity co-vary during transition of arousal states and associations between cortical and autonomic activity are better reflected by HRV (26).

Cognitive dysfunction is one of the common co-morbidities that occur in epilepsy, including memory, attention, and processing difficulties (27). Recent studies have shown evidence for the active involvement of alpha activity in cognitive processes (28, 29) through its active role in the control of neural excitability in the cortex by top-down modulation of neural activity in cortical networks (30, 31).

In the present work, we evaluated the association between EEG oscillatory behavior and autonomic function in patients with refractory epilepsy. Our results show that in RE patients, there is a strong positive correlation between HRV and the power of cortical oscillatory cortical activity in all studied EEG bands (delta, theta, alpha, and beta) in all regions of interest in both hemispheres, the opposite pattern found in controls which had low or negative correlation between these variables.

We observed a shift to the left in alpha peak frequency (APF) in RE patients. Other studies showed that this decrease could be associated with the continuous use of antiepileptic drugs (AEDs) (32). However, Pyrzowski et al. (33) showed that alpha rhythm abnormalities in people with epilepsy had weak or non-significant dependences on the number of AEDs taken by the patients. An earlier study by Larsson (34) also showed that patients with refractory epilepsy had APF around 9 Hz in the temporal and occipital regions. A recent study of the effects of prolonged social isolation and spatial confinement (35) showed a reduction of APF during isolation which the authors associate with a reduced vigilance state and sensory deprivation. These data corroborate the literature and that the APF shift of alpha 1 may be associated with cognitive problems and memory (33, 36).

Previous works had compared the resting-state EEG power spectrum in people with epilepsy with that of healthy subjects. For instance, while Pellegrino et al. (37) showed no difference between groups after Bonferroni correction, Ricci et al. (38) found differences only in theta power, which was lower in people with epilepsy. Another study by Croce et al. (39) deployed machine learning tools to predict the clinical response to anti-seizure medications using the resting-EEG recordings of people with temporal lobe epilepsy. The authors found they could predict the clinical response to an anti-seizure medication (levetiracetam), from the patients' EEG phenotype. However, different from the present work, the experimental group in the above studies is composed of non-refractory epilepsy patients.

Our findings highlight the distinctive coupling between cardiac parameters and cortical electrical activity in all EEG bands of RE patients when compared to the control group. In the control group, on the other hand, the correlation between these parameters was either low or negative in all ROIs both in the left and the right hemispheres. SD1/RMSSD is the primary time-domain measure of tonic vagal activity (20). Our univariate Spearman correlation and multivariate linear regression analysis showed essentially that a linear model does not seem appropriate to establish a robust causality between EEG channel-specific power and HRVs. The correlation between EEG power and RMSSD in RE patients could underlie a process aimed at restoring the homeostatic equilibrium of cortical networks disrupted by epileptogenesis. There are some therapeutic approaches that have been proposed to provide neuronal networks with the means necessary to restore homeostatic balance in people with epilepsy (40, 41).

The results of our HEP analyses provide additional support for the involvement of cardiac variables in the maintenance of a dysfunctional set point in the epileptic brain. Our data showed that the amplitude of HEP in widespread locations over the scalp is higher in RE patients than in the control group, probably reflecting a more robust phase-locked activation of cortical neurons associated with the cardiac event (42). heartbeat-related modulations in cortical activity are connected to fluctuations of interoceptive attention and spontaneous shifts between interoception and exteroception (HEP amplitudes) (43). The difference in HEP amplitude modulation between RE patients and controls may underlie different processing of interoceptive signals. Other studies show HEP amplitude is positively correlated with arousal levels and may be related to a state of greater physiological activation (reactivity of the sympathetic system) (10, 44).

Taken together, the present results point to a significant alteration in heart-brain interaction in patients with refractory epilepsy. Previous studies had already shown that epilepsy affects cardiac function and impacts on the clinical course and prognosis of patients, causing arrhythmias and sudden unexpected death in epilepsy (SUDEP) (45). Our study highlights how neurovisceral integrative networks can change in epilepsy patients with possible reverberations in cognitive networks.

### Limitations and future research

There were several limitations to this work that need to be considered. Most important, the sample size of both groups is small and lacked statistical power to do more robust analyses or detect smaller effects. Future studies should include larger, possibly more representative samples of epilepsy patients. However, we hope future studies will validate and extend the preliminary findings we reported. The COVID-19 pandemic hampered our efforts to recruit more subjects and perform additional experiments.

Future research directions should evaluate neurovisceral interactive networks during performance of cognitive or emotional tasks. These studies could help better characterize the implications of network disturbances caused by epilepsy on patient's cognitive and emotional capabilities.

## Data availability statement

The raw data supporting the conclusions of this article are not publicly available due to privacy reasons but are available from the corresponding author on reasonable request.

## Ethics statement

The studies involving human participants were reviewed and approved by the Research Ethics Committee of The Federal University of Para. The patients/participants provided their written informed consent to participate in this study.

## Author contributions

AP conceived the study. AP, EM, FG, TR, and RN performed the experiments. AP, EM, TR, RN, and BD analyzed the data. All authors contributed to the article and approved the submitted version.

## Funding

This work was supported by CNPQ (Research Productivity 312060/2020-3 for AP and Universal MCTIC/CNPq 436717/2018-2 for BD) and UFPA/PAPQ.

## Conflict of interest

The authors declare that the research was conducted in the absence of any commercial or financial relationships that could be construed as a potential conflict of interest.

## Publisher's note

All claims expressed in this article are solely those of the authors and do not necessarily represent those of their affiliated organizations, or those of the publisher, the editors and the reviewers. Any product that may be evaluated in this article, or claim that may be made by its manufacturer, is not guaranteed or endorsed by the publisher.
